# MHC Class I Antigen Presentation of DRiP-Derived Peptides from a Model Antigen Is Not Dependent on the AAA ATPase p97

**DOI:** 10.1371/journal.pone.0067796

**Published:** 2013-07-02

**Authors:** Amy L. Palmer, Brian P. Dolan

**Affiliations:** Department of Biomedical Sciences, Oregon State University, Corvallis, Oregon, United States of America; Johns Hopkins University, United States of America

## Abstract

CD8^+^ T cells are responsible for killing cells of the body that have become infected or oncogenically transformed. In order to do so, effector CD8^+^ T cells must recognize their cognate antigenic peptide bound to a MHC class I molecule that has been directly presented by the target cell. Due to the rapid nature of antigen presentation, it is believed that antigenic peptides are derived from a subset of newly synthesized proteins which are degraded almost immediately following synthesis and termed Defective Ribosomal Products or DRiPs. We have recently reported on a bioassay which can distinguish antigen presentation of DRiP substrates from other forms of rapidly degraded proteins and found that poly-ubiquitin chain disassembly may be necessary for efficient DRiP presentation. The AAA ATPase p97 protein is necessary for efficient cross-presentation of antigens on MHC class I molecules and plays an important role in extracting mis-folded proteins from the endoplasmic reticulum. Here, we find that genetic ablation or chemical inhibition of p97 does not diminish DRiP antigen presentation to any great extent nor does it alter the levels of MHC class I molecules on the cell surface, despite our observations that p97 inhibition increased the levels of poly-ubiquitinated proteins in the cell. These data demonstrate that inhibiting poly-ubiquitin chain disassembly alone is insufficient to abolish DRiP presentation.

## Introduction

In order to eliminate cells that have become infected or transformed, CD8^+^ T cells first need to be primed to the presence of disease-associated antigens and then must recognize the peptide-antigen bound to the cognate MHC class I molecule on the surface of the cell targeted for elimination. T cell priming is due in large part to dendritic cell (DC) cross-presentation of antigens whereby DCs engulf proteins and degrade them into antigenic peptides prior to loading of these peptides onto MHC class I molecules. DC cross-presentation is a highly efficient process, where very low levels of antigenic substrates can give rise to sufficient peptide-MHC complexes in order to stimulate CD8^+^ T cells [1], [2], [3].

In contrast to cross-presentation, direct antigen presentation occurs in the body’s own cells that are surveyed by primed-CD8^+^ T cells hunting for their cognate peptide antigen. The direct presentation of peptide antigens derived from viral or self-proteins occurs quite rapidly following polypeptide synthesis [4], [5], [6], [7]. This rapid presentation of peptides from metabolically-stable proteins gave rise to the Defective Ribosomal Products (DRiP) hypothesis [8] which postulates that a subset of newly synthesized proteins were in some way defective and would be quickly removed from the cell to prevent the build-up of detrimental, mis-folded proteins. Like cross-presentation, DRiP antigen presentation is a highly efficient process [9], [10], [11], which is advantageous for the immune response: virally infected cells can be detected and eliminated before the infection spreads and cancerous cells could display antigenic peptides derived from a relatively small pool of tumor-specific proteins. Because both DRiP presentation and cross-presentation are remarkably efficient, we have speculated that some elements of the two presentation pathways may overlap [12].

We have recently described a cell-based system that allows us to measure efficient presentation of peptides specifically from DRiP substrates [10], [13]. We also identified chemical inhibitors of DRiP antigen presentation which did not diminish presentation of peptides derived from normal protein turnover. Interestingly, both inhibitors increased levels of poly-ubiquitin conjugated proteins within the cell. One compound, Eeyrstatin I (Eer1) is also known to inhibit the process of ER-associated degradation (ERAD), the metabolic pathway by which unfolded proteins in the ER are translocated to the cytosol for degradation by the proteasome [14]. The ERAD pathway is also employed by DC during cross-presentation [15], [16], [17], presumably to remove endocytosed antigens from phagocytic vesicles to allow proteasome mediated degradation followed by peptide loading and presentation.

Both cross-presentation [16], [17], [18] and ERAD [19] rely on the AAA ATPase p97. Inhibition of p97 also leads to an increase in levels of poly-ubiquitinated proteins in cells [19], [20], [21]. In addition, p97 is also known to associate with the proteasome [22], [23]. Because many of these cellular functions are known or hypothesized to be related to DRiP presentation, we wished to determine what role, if any, p97 would have in DRiP antigen presentation. We find, however, that genetic and chemical inhibition of p97 did not alter presentation of peptide antigens from DRiP substrates, but did increase levels of poly-ubiquitinated proteins within the cell. Though p97 does not appear to be involved, these data demonstrate that alteration of poly-ubiquitin profiles alone does not diminish DRiP presentation, rather specific molecular pathways governing ubiquitin remodeling are likely to be responsible for efficient antigen presentation.

## Materials and Methods

### Cell Lines and Antibodies

EL4 and EL4 cells stably expressing shield-controlled recombinant antigenic protein (here after EL4/SCRAP) have been previously described [10] and were cultured in RMPI 1640 supplemented with 10 mM HEPES, 20 mM Glutamax, and 7.5% fetal calf serum (all from Life Technologies) at 37°C in 6% CO_2_. JY cells were a gift from Drs. Jack Bennink and Jonathan Yewdell at National Institutes of Health [24]. Rabbit anti actin antibodies were from Bethyl Laboratories Inc, anti-p97 monoclonal antibody (clone 58.13.3) was from Fitzgerald Industries International, anti-poly ubiquitin monoclonal antibody (clone FK2) was from Enzo, anti-MHC class I K^b^ monoclonal antibody (clone Y3), anti-HLA A,B,C (clone W6/32), and monoclonal antibody 25D-1.16 (anti K^b^-SIINFEKL) were gifts of Drs. Bennink and Yewdell (NIH). The PE-Cy5.5 coupled anti-Thy1.1 (and corresponding isotype control antibody) were from eBioscience, and DyLight 649-coupled goat anti-mouse IgG was from from KPL. Seconday antibodies for western blot analysis, IRDye 680LT goat anti-mouse and IRDye 800CW anti-rabbit polyclonal antibodies, were from LI-COR. 25D-1.1.6 and Y3 antibodies were directly coupled to Alexa 647 dye using a Molecular Probes protein labeling kit and following the manufactures instructions.

### Transfections

The IRES containing vector pMSCV Thy1.1 expressing wild-type or dominant negative (DN) p97 and Thy1.1 were from Dr. Peter Cresswell (Yale) and have been previously described [17]. The ERAD substrate TCRα-GFP in pLNCX2 was from Dr. Yihong Ye and has been previously described [25]. Transfections were performed with an Amaxa 96-well shuttle nucleofector (Lonza). Briefly, 5×10^5^ EL4/SCRAP cells were resuspended in 20 µl transfection solution SF to which 300 ng of DNA was added and cells placed in one well of the cuvette plate. Cells were transfected using program DS-113, incubated for 5 minutes at 37°C, and then plated in complete media.

### Antigen Presentation Assays

Cells were chilled on ice for 10 minutes and resuspended in ice-cold citric acid buffer (0.13 M citric acid and 0.0625 M dibasic sodium phosphate, pH = 3) at 1–2×10^7^ cells/ml for 2 minutes. Ice cold RPMI 1640 was added to wash the cells and then cells were resuspended in warm tissue culture media at 1×10^6^ cells/ml. Cells were cultured for indicated times in the presence of 5 µM shield-1 and harvested for flow cytometry analysis. In some experiments, *N^2^,N^4^*-dibenzylquinazoline-2,4-diamine (DBeQ) was added to cells following acid wash at the indicated concentrations. DBeQ was a kind gift of Dr. Tsui-Fen Chou (UCLA).

### Flow Cytometry

Cells were harvested and washed in cold Hank’s Balanced Salt solution (HBSS, Life Technologies) supplemented with 0.1% BSA (Amresco). For K^b^-SIINFEKL and Thy1.1 expression, cells were stained with Alexa-647 coupled 25D-1.16 mAb and anti Thy1.1 antibody for 30 minutes at 4°C, washed once and resuspended in HBSS/BSA. For total MHC class I analysis, cells were first stained with either Y3 or W6/32 antibody, washed, and then stained with DyLight 649-coupled goat anti-mouse IgG for 30 minutes at 4°C followed by washing with HBSS/BSA. In some experiments cells were stained with Y3 directly coupled to Alexa 647 for 30 minutes on ice followed by HBSS/BSA washing. Cells were then analyzed for expression of GFP and fluorescent antibody binding by flow cytometry using an Accuri C6 flow cytometer (BD Biosciences). Samples were analyzed using the BD Accuri C6 software. For kinetic measurement’s, the mean fluorescence intensity (MFI) levels of both GFP and K^b^-SIINFEKL at time 0 hours were treated as background and subtracted from the MFI levels at later time points.

### Toxicity Tests

Cells were treated with varying concentrations of DBeQ or DMSO alone and cultured for 4 hours. At that time toxic effects of the drugs were determined using the alamarBlue™ viability assay (Invitrogen) with a few modifications to the manufacturer’s protocol. Briefly, cells were harvested and washed in cold Hank’s Balanced Salt solution (HBSS, Life Technologies) supplemented with 0.1% BSA (Amresco). Cells were incubated with alamarBlue™ for 30 minutes at 4°C and fluorescence of substrate was measured using a Tecan Infinite 200 microplate reader.

### Western Blot Analysis

Cells were harvested and resuspended to 10^7^cells/ml in SDS-Sample buffer (Amresco) containing 10 nM N-Ethylmaleimide (Alpha Aesar) and immediately boiled for 20 minutes at 95°C. An equal volume of water supplemented with 10 mM DTT was added to the lysate and boiled for an additional 10 minutes. Samples were then resolved by SDS-PAGE analysis and transferred to nitrocellulose membranes (LI-COR). Membranes were blocked with a 4% dehydrated milk solution made in Tris-buffered saline with 0.1% Tween 20 (TBS-T) for 1 hour. Antibody solutions in 0.5% milk/TBS-T were then added to membranes and incubated with agitation for 1 hour at room temperature. Membranes were washed with TBS-T for 10 minutes, then rinsed with water and analyzed using an Odyssey infrared imaged (LI-COR). Signals were quantified using the instrumental software.

### Statistics

Student’s t-test analysis and linear regression calculations were performed using GraphPad Prism software.

## Results

### Genetic Ablation of p97 does not Alter DRiP Antigen Presentation

We have recently described a method for distinguishing DRiP antigen presentation from presentation of peptides derived from substrates as the result of normal protein turnover [10], [13]. The system took advantage of the mutant form of FKBP12 which destabilizes the protein to which it is appended, a process which can be reversed with a small molecule termed shield-1 [26]. The shield-controlled recombinant antigenic protein (or SCRAP) contains the destabilization domain, followed by the mouse MHC class I K^b^-binding SIINFEKL peptide and GFP. When EL4 cells stably expressing SCRAP were exposed to saturating doses of shield-1, robust presentation of SIINFEKL continued despite the biochemical evidence that all synthesized SCRAP was spared from degradation [10]. This observation indicated a small fraction of SCRAP DRiPs were responsible for providing peptides for presentation. Furthermore the presentation of peptides from the shield-1 insensitive SCRAP DRiPs could be modulated by certain chemical inhibitors which did not prevent presentation of other forms of SCRAP.

**Figure 1:**
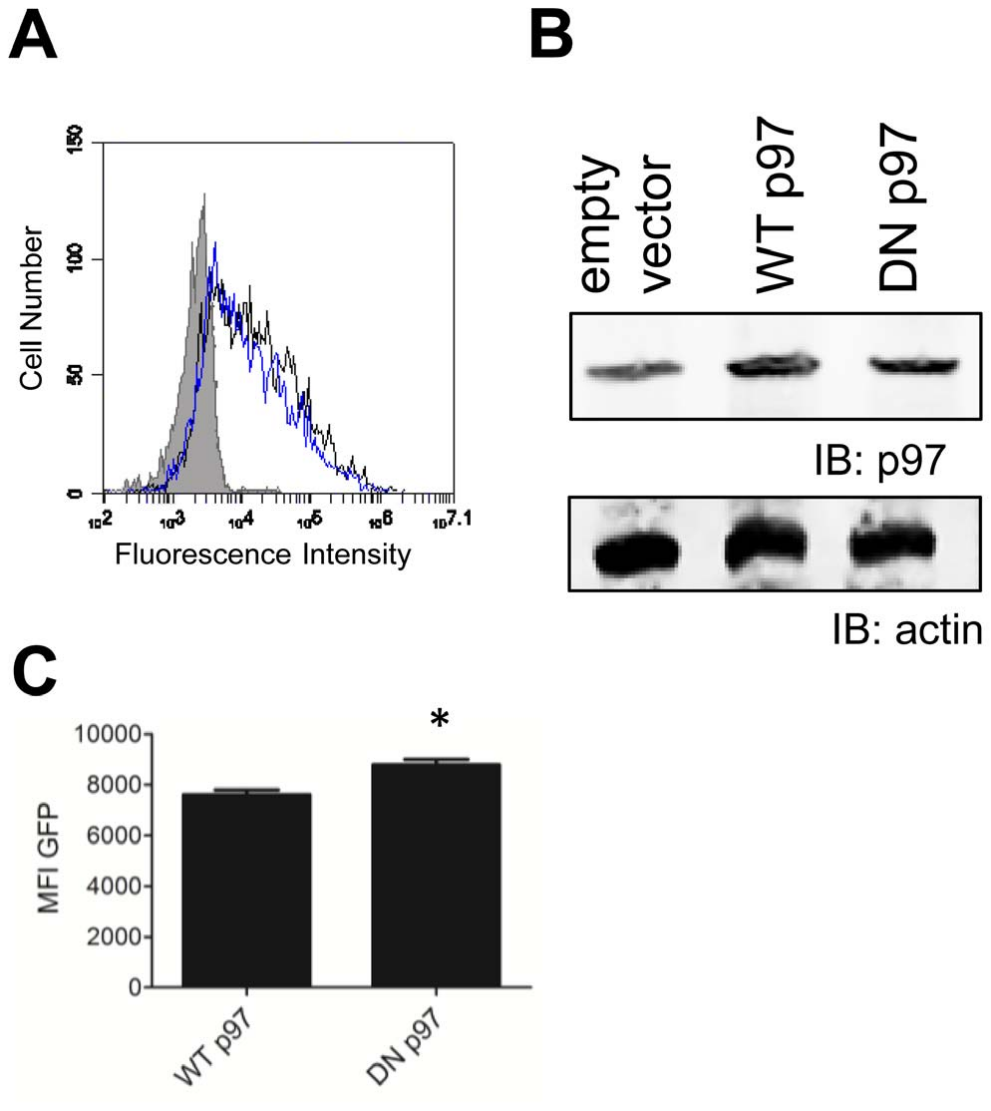
Transfected EL4/SCRAP cells express elevated levels of p97 protein. *A*. EL4/SCRAP cells were transfected with pMSCV containing an IRES insert that allowed for dual expression of Thy1.1 and either wild type (black trace) or a dominant-negative mutant (blue trace) of p97. Cells were stained with anti-Thy1.1 antibody 24 hours post transfected and analyzed by FACS. Representative histograms are shown with the isotype control antibody staining represented by the shaded histogram. *B*. Total cell lysates from cells transfected with wild type (WT) or dominant negative (DN) p97 vectors were prepared 24 hours after transfection and resolved by SDS-PAGE followed by western blot analysis for p97 protein and actin. *C.* EL4 cells were transfected with constructs encoding a mutated TCRα- GFP construct and either WT or DN expression plasmids. The following day Thy1.1 cells were analyzed for GFP expression. DN transfected cells had elevated levels of GFP (p<0.05) indicating inhibited ERAD function.

One compound which repressed presentation of the DRiP form (ie shield-1 insensitive) of SCRAP inhibits the AAA ATPase p97 [14], [27]. To determine if p97 was necessary for DRiP antigen presentation, we transfected EL4/SCRAP cells with vectors containing either wild type or the dominant negative (DN) form of p97 expressed in an IRES construct that allows simultaneous expression of cell-surface Thy1.1 [17]. As shown in figure 1A >50% of cells expressed Thy1.1 following transfection and elevated levels of p97 protein were detected in western blot lysates (figure 1B). To determine if the DN p97 was functioning in EL4 cells, we tested the ability of transfected cells to rescue expression of mutant TCRα-GFP, encoded on a second transfected plasmid. This construct is subject to p97-dependant ERAD and inhibition of ERAD results in accumulation of GFP [25]. As anticipated, expression of DN p97 led to an increase in GFP signal (p<0.05) indicating that DN p97 expression in EL4 cells does inhibit p97-dependant cellular processes.

**Figure 2:**
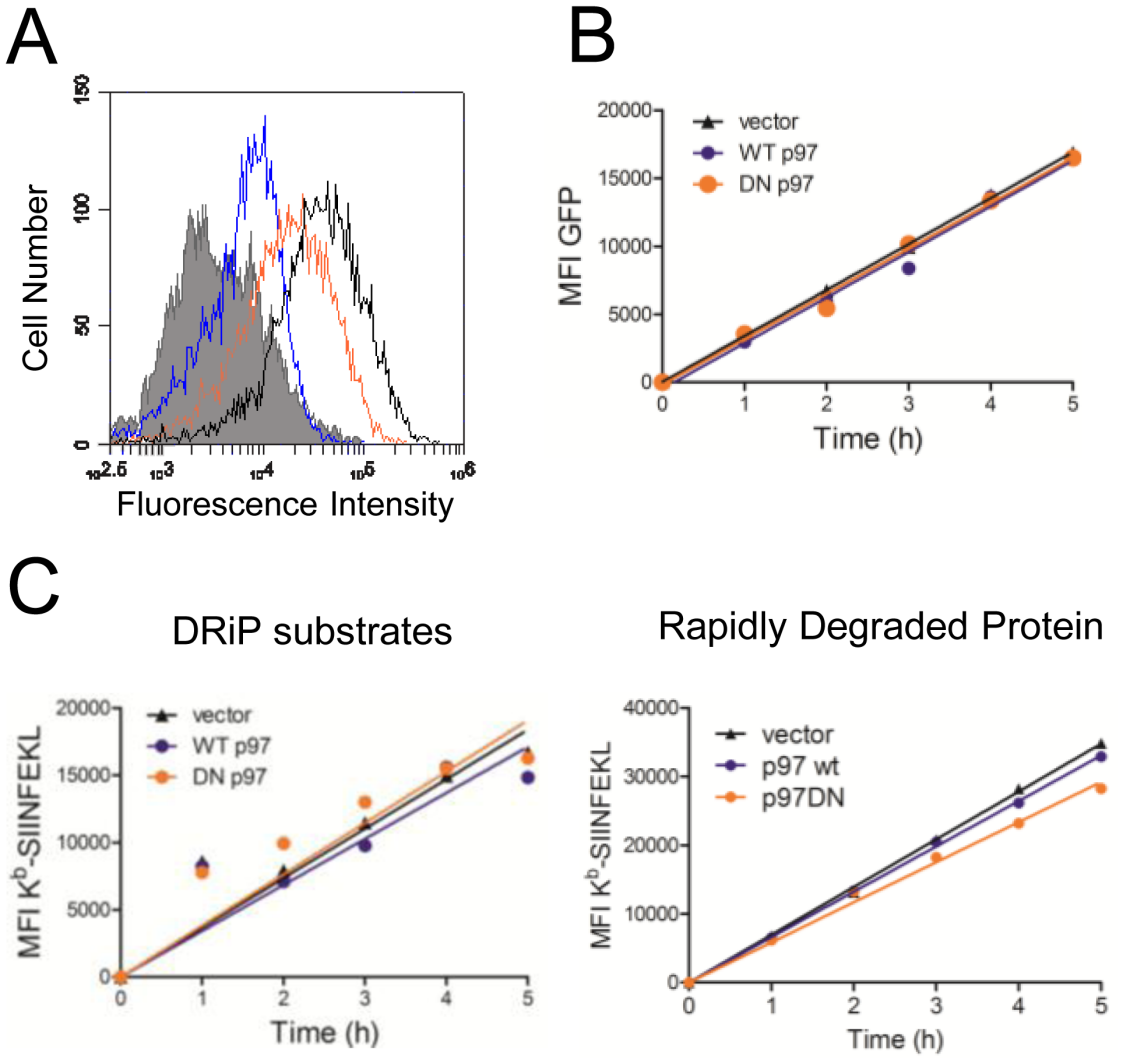
Genetic ablation of p97 does not diminish DRiP antigen presentation. *A.* EL4/SCRAP cells were placed in ice-cold citric acid buffer (pH 3) for 2 minutes, washed, re-suspended in tissue culture media, and cultured for 5 hours in the presence of shield-1 before staining with the monoclonal antibody 25D-1.16 to detect K^b^-SIINFEKL complexes at the cell surface. K^b^-SIINFEKL complexes recovered from near background levels (blue trace) to approximately 50% (orange trace) the level of cells that had not been washed in acid (black trace). EL4 cells that do not express K^b^-SIINFEKL are shown as a negative control (shaded histogram) and are considered background 25D- 1.16 staining. *B and C.* EL4/SCRAP cells were transfected with either an empty vector, wild type (WT) or DN p97 constructs and 24 hours later, washed in mild citric acid to elute existing peptides from MHC class I molecules. Cells were then treated with 5 µM shield-1 and cultured for 5 hours. At indicated times cells were collected and analyzed by FACS. Cells expressing Thy1.1 were subsequently analyzed for GFP expression (*B*) and the mean fluorescence intensity (MFI) plotted. K^b^-SIINFEKL expression (*C*) was analyzed for both DRiP substrates (left) and the rapidly degraded form of SCRAP (right) and the MFI plotted as a function of time.

To determine if p97 is necessary for successful antigen presentation, it is necessary to treat EL4/SCRAP cells with cold citric acid to remove existing peptide-MHC complexes from the cell surface. As shown in figure 2A, acid washing removes almost all K^b^-SIINFEKL complexes from EL4/SCRAP cells as determined by staining with the K^b^-SIINFEKL specific monoclonal antibody 25D-1.16. Immediately following acid washing, 25D-1.16 staining of EL4/SCRAP cells is similar to EL4 cells which do not generate K^b^-SIINFEKL complexes. Following 5 hours of culture in the presence of shield-1, K^b^-SIINFEKL levels have recovered to approximately half the levels measured in non-treated cells (Figure 2A), in agreement with our previously published results [10]. To determine the effect of p97 inhibition on antigen presentation, we transfected cells with p97 expression plasmids (or empty vector controls) and measured K^b^-SIINFEKL recovery in acid-washed transfectants in the presence of a saturating dose (5 µM) of shield-1 for a 5 hour time period. Cells were harvested every hour for five hours and analyzed by flow cytometry for SCRAP synthesis and DRiP antigen presentation by gating on Thy1.1 positive cells. DN p97 expression did not affect either GFP accumulation in cells (figure 2B) nor did it alter the presentation of the SIINFEKL peptide from DRiP forms of SCRAP (figure 2C). Inhibition of p97 did not impact the recovery of K^b^-SIINFEKL complexes in the absence of shield-1 (Figure 2C), where all expressed copies of SCRAP are subject to rapid degradation. Therefore, genetically inhibiting the function of p97 does not alter DRiP presentation or substrate synthesis.

### Inhibition of p97 Leads to an Increase in Poly-ubiquitinated Proteins

**Figure 3:**
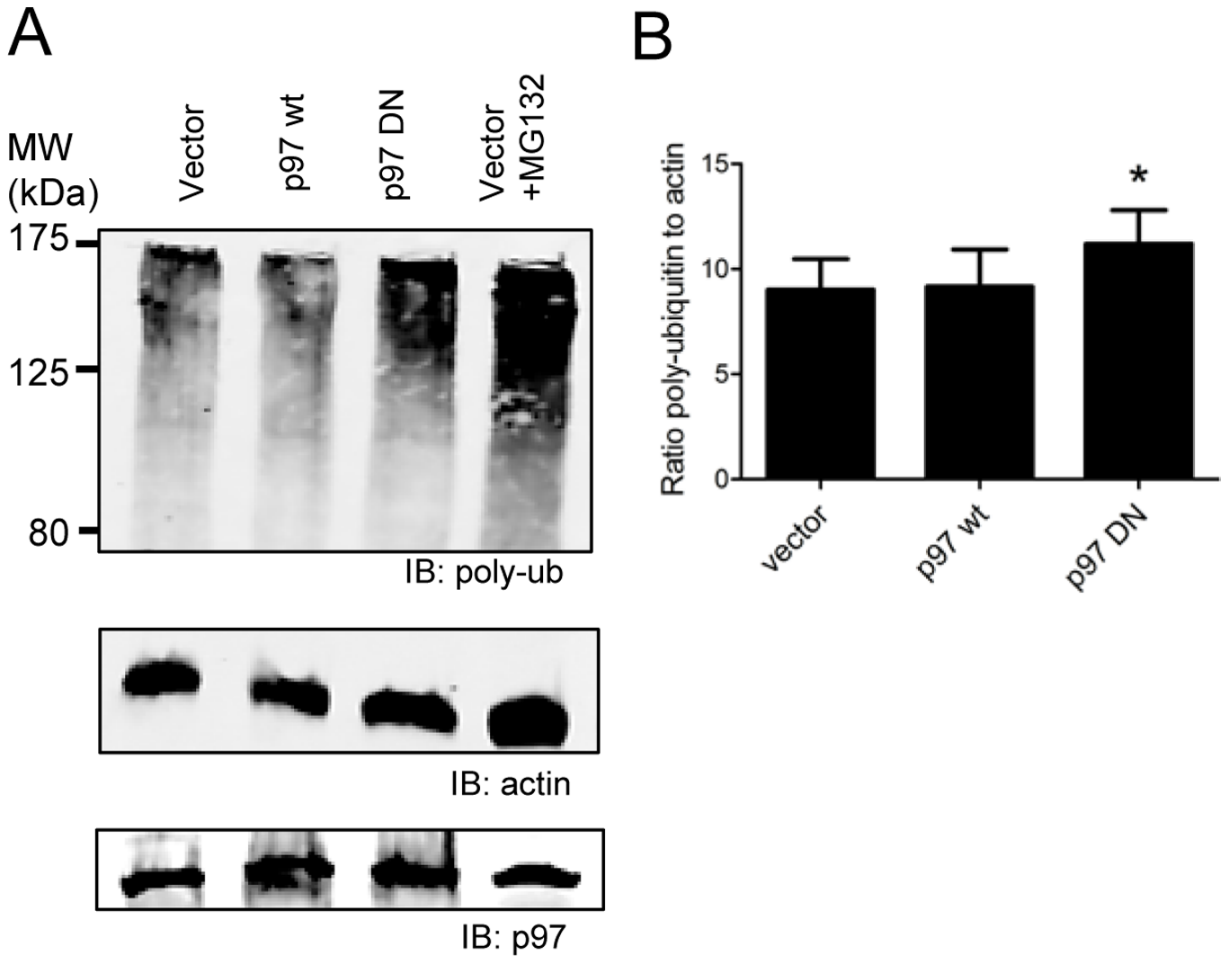
Poly-ubiquitinated proteins accumulate in cells expressing DN p97. Total cell lysates were prepared 24 hours after transfection with empty vector, wild type p97, or DN p97 protein and analyzed by SDS-PAGE and western plot analysis. *A.* Blots were stained with anti-poly-ubiquitin monoclonal antibodies (top), anti-actin polyclonal antibodies (middle), or anti-p97 monoclonal antibodies (bottom). *B.* Signals of both poly-ubiquitin conjugated protein were normalized to actin levels in the cell and are depicted graphically. The average ratio of poly-ubiquitin signal to actin for four experiments is plotted. DN p97 expression resulted in a statistically significant accumulation of poly-ubiquitin conjugates in cells (p<0.05).

Our previous work demonstrated that chemical inhibition of DRiP presentation was accompanied by an increase in the level of poly-ubiquitinated proteins in cells. A similar increase in poly-ubiquitinated proteins accompanies p97 ablation presumably due to the accumulation of ubiquitinated ERAD substrates that are unable to be degraded [19], [20], [21]. To determine if the DN p97 construct functioned in a similar manner in our system, we examined total cell lysates from EL4/SCRAP cells transfected with the empty vector, wild type p97 or the DN mutant of p97 for levels of poly-ubiquitinated proteins. As shown in figure 3, DN p97-expressing cells appear to have elevated levels of poly-ubiquitin protein conjugates which was confirmed when the poly-ubiquitin signal was normalized to the levels of actin in the cell lysate (p<0.05). Therefore, expression of DN p97 does in fact inhibit p97 functions in these cells.

### Chemical Inhibition of p97 does not Alter DRiP Antigen Presentation

**Figure 4:**
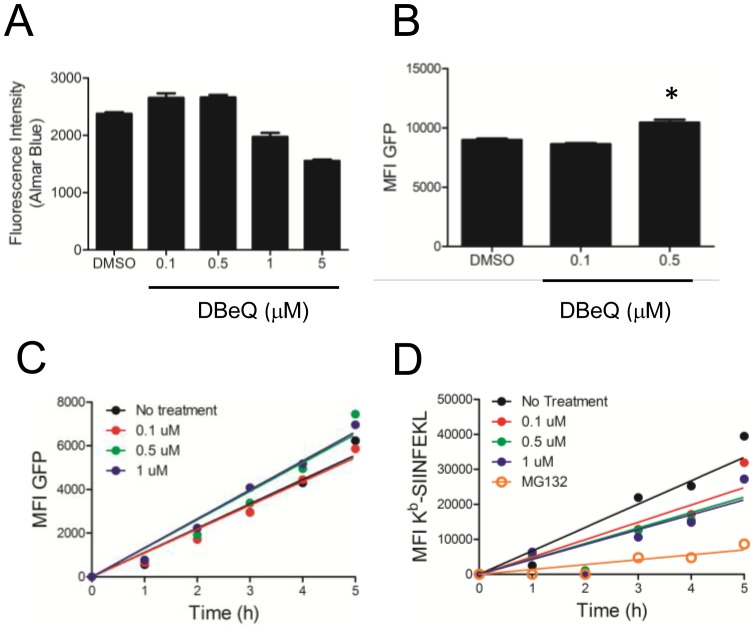
DBeQ treatment fails to inhibit DRiP antigen presentation. *A*. Cells were tested for metabolic turnover of alamarBlue four hours post DBeQ treatment as a proxy for toxicity. Concentrations of DBeQ >1 µM showed single-dose toxic effects. *B.* DBeQ treatment resulted in accumulation of mutant TCRα-GFP at non-toxic doses of DBeQ (* p<0.05). Acid-washed EL4/SCRAP cells were treated with 5 µM shield-1 and increasing amounts of the p97-inhibiting compound DBeQ or 10 µM MG132. At indicated times, cells were analyzed for either GFP expression (*C*) and K^b^-SIINFEKL accumulation (*D*).

A recently described compound, *N^2^,N^4^*-dibenzylquinazoline-2,4-diamine, also known as DBeQ, was shown to inhibit p97 function in cells [28]. In EL4 cells, DBeQ exhibited single-dose toxicity at concentrations greater than 0.5 µM (Figure 4A). To determine if DBeQ treatment inhibited p97 function we again assayed for rescue of the GFP-coupled ERAD substrate. EL4 cells expressing the mutant TCRα-GFP construct had increased GFP fluorescence when treated with 0.5 µM DBeQ (p<0.05, Figure 4B) similar to expression of DN p97. Therefore, treating EL4 cells with a sub-toxic dose of DBeQ can inhibit p97 function. We then tested DBeQ to determine if it could inhibit DRiP antigen presentation. Treatment with increasing concentrations of DBeQ showed a minor inhibition of DRiP antigen presentation (figure 4D) and no noticeable effect on SCRAP synthesis (figure 4C). However the effect on antigen presentation was much more mild than proteasome inhibition and did not demonstrate a clear dose-dependent response. Therefore, it is unlikely that chemical inhibition of p97 with DBeQ significantly affects DRiP antigen presentation.

### Inhibition of p97 does not Alter MHC Class I Levels

**Figure 5:**
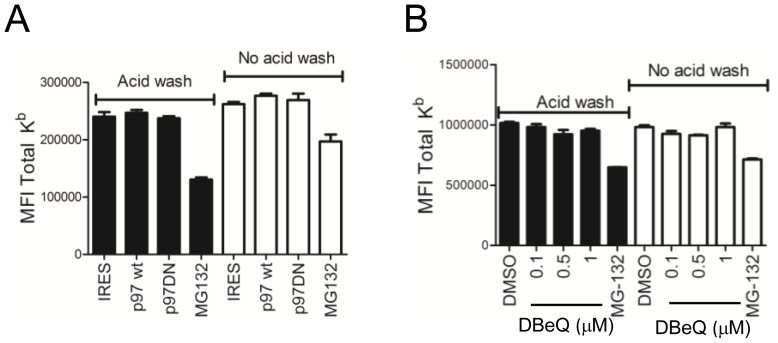
Inhibition of p97 does not diminish over all levels of cell-surface MHC class I. *A*. EL4 cells were transfected with p97 constructs and analyzed 24 hours later for cell-surface K^b^ expression by FACS by gating onThy1.1 cells. Cells were either left untreated (white bars) or washed in citric acid buffer (black bars) and allowed to recover for five hours before analysis. The staining was done in triplicate. *B.* EL4 cells were treated with indicated levels of DBeQ for 5 hours and analyzed by FACS for cell-surface K^b^ expression. No consistent statistical difference at any concentration of DBeQ occurred between experiments, though MG132 did statistically diminish K^b^ levels (p<0.05). In both experiments, EL4 cells treated with 10 µM MG132 for 5 hours was included as a positive control for inhibiting antigen presentation and diminishing cell-surface K^b^.

**Figure 6:**
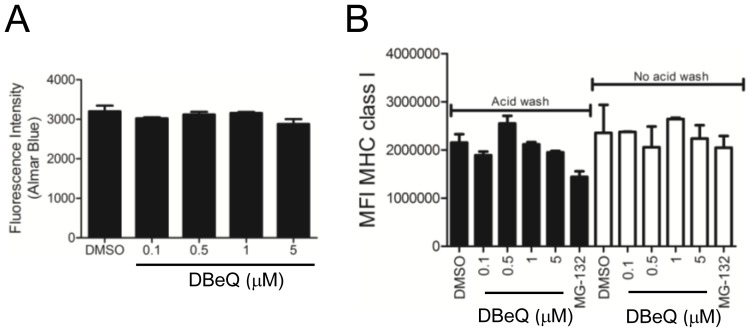
DBeQ treatment of JY cells does not diminish HLA class I levels. *A.* AlamarBlue fluorescence of JY cells treated with either DMSO or increasing doses of DBeQ demonstrates JY cells are more resistant to the single-dose toxic effects of DBeQ. *B.* JY cells were either washed in citric acid buffer (black bars) or left untreated (white bars) and stained, in triplicate, 5 hours post treatment with indicated concentrations of DBeQ or MG132. Cells were analyzed for total MHC class I expression using the monoclonal antibody W6/32 which detects HLA A,B, and C molecules. This experiment is representative of three independent experiments and no statistical difference between DMSO and DBeQ treated cells was noted.

While our data thus far suggest that p97 is dispensable for DRiP antigen presentation, this may only hold true for our specific construct and not be generalized to other DRiP substrates. We therefore measured total K^b^ on the surface of EL4 cells that were either untreated or washed in citric acid to remove existing MHC class I-peptide complexes expressing DN p97 or wild type p97 proteins. As shown in figure 5A, 24 hours after transfection with p97 constructs K^b^ levels on the cell surface were unaltered. We also determined the effect of a 5 hour DBeQ treatment and found total cell-surface K^b^ levels appear to be slightly diminished, though any statistical difference noted at a particular concentration was not consistent between the three experiments that were conducted (figure 5B). In contrast a 5 hour treatment with 10 µM MG132 reduced K^b^ levels by almost 60–80% by inhibiting proteasome function and preventing peptide generation (p<0.05). To confirm this observation and to account for the toxic effect of DBeQ in EL4 cells, we tested the effect of DBeQ treatment in a second cell line. The human lymphoblastoid cell line JY was treated with increasing concentrations of DBeQ without diminishing the conversion of alamarBlue (Figure 6A). A five hour treatment with DBeQ did not alter the expression of MHC class I on JY cells that had been treated with citric acid or left untreated. The data is an average of triplicate staining and the experiment was repeated three times. No statistical difference was noted at any concentration of DBeQ treatment. Therefore p97 function is not necessary for antigen presentation.

## Discussion

The AAA ATPase p97 activity has been reported for several cellular pathways which are likely involved in DRiP antigen presentation. Studies have found p97 directly linked to the proteasome [22], [23], and is an essential player in the degradation of mis-folded proteins, particularly those associated within membrane-bound organelles such as the ER and autophagosomes [29], [30], [31]. Successful cross-presentation of antigens on MHC class I molecules also requires functional p97 [16], [17], [18]. Additionally, a known chemical inhibitor of DRiP presentation targets p97 [27]. The circumstantial evidence certainly pointed to p97 playing an important role in DRiP presentation, however the data reported here suggest this is not the case. Both genetic ablation and chemical inhibition of p97 did not impact DRiP presentation from a model substrate and did not have an overall impact on the levels of cell-surface MHC class I.

DRiP antigen presentation is an efficient process, which allows cells of the body to ensure that peptides are presented from relatively few substrates assuring successful immune surveillance of rare transcripts. Likewise, the cross-presentation of peptides from engulfed antigens is also highly efficient: peptide-MHC complexes can be generated in sufficient quantities to stimulate CD8^+^ T cells from a relatively small input of substrates. Because of the high efficiency in both DRiP presentation and cross-presentation, overlap between the two processes is likely to occur [12]. However, the cellular locations of the two processes are sufficiently different that overlap may be more rare than previously hypothesized. Cross-presentation first requires antigenic protein to be endocytosed into the cell prior to degradation either in the cytosol or endosomal compartments [32], [33], [34]. DRiPs form as the result of protein synthesis, a process that occurs on ribosomes located in the cytosol or on the cytosolic face of the ER. SCRAP does not contain an ER-targeting sequence, and while such a sequence is not a prerequisite for synthesis on ER-bound ribosomes [35], [36], it is highly unlikely that SCRAP, either as a DRiP or functional protein, would ever be found in the ER. It is therefore unlikely that the cellular processes for extraction of endocytosed antigens would be involved in SCRAP presentation. However, this does not explain why p97 inhibition did not generally lower the levels of cell-surface MHC class I molecules, as presumably some DRiPs would localize within the ER and be subject to p97 mediated ERAD.

Data presented here also demonstrate that DRiP presentation can continue unabated while poly-ubiquitin chain disassembly is prevented. Previous work demonstrated that inhibitors of DRiP presentation also increased the levels of poly-ubiquitin conjugated proteins in the cell. We speculated that one or more deubiquitinating enzymes (or DUBs) may be responsible for DRiP presentation. However, we did not rule out that simply altering ubiquitin homeostasis within the cell could adversely impact presentation of DRiP-derived peptides. In this study, like many others, p97 inhibition also resulted in increased levels of poly-ubiquitin conjugated proteins. Therefore, simply altering the levels of poly-ubiquitinated proteins in the cell (and presumably, altering the homeostasis of ubiquitin) is not sufficient to inhibit DRiP presentation and lends credence to the hypothesis that specific DUBs are responsible for ensuring presentation of peptides from DRiPs.

Recent studies have suggested that antigen presentation is compartmentalized [13], [37], [38], though no work to date has identified such a compartment. As p97 has an important role in removing or remodeling proteins in diverse organelles including the nucleus, mitochondria, ER, lysosomes, autophagosomes, golgi and probably more, our findings would suggest the antigen presentation compartment may not have a higher order structure similar to an organelle. It is possible that such a compartment is rather a microdomain in the cell where all the necessary molecules come into contact to perform the function of antigen presentation. If so, further examination of molecular pathways that govern DRiP presentation should shed light on how a subset of newly synthesized proteins can be degraded and loaded onto MHC class I molecules in a highly efficient manner.
